# Safety and Efficacy of Laser Versus Cryotherapy in the Treatment of Lentigines: A Systematic Review and Meta-analysis of Randomized Controlled Trials: Short title: Safety and Efficacy of Laser Versus Cryotherapy in the Treatment of Lentigines

**DOI:** 10.31661/gmj.v15i.4137

**Published:** 2026-03-02

**Authors:** Mohammad Almohideb

**Affiliations:** ^1^ College of Medicine, King Saud Bin Abdulaziz University for Health Sciences, Riyadh, Saudi Arabia; ^2^ King Abdullah International Medical Research Center, Riyadh, Saudi Arabia

**Keywords:** Lentigines, Laser, Cryotherapy

## Abstract

**Background:**

Lentignies is one of the pigmentary skin lesions causing abnormal skin color in sun-exposed areas in particular the hands and the face. In this systematic review and meta-analysis, we aimed to study the safety and efficacy of cryotherapy versus laser in lentigines treatment.

**Materials and Methods:**

Beginning from inception and up to 19th May 2023, we did a comprehensive search strategy including five databases (Web of Science=33, Scopus=79, PubMed=28, Virtual Health Library=30 and Scholar=622). The search strategy was performed according to the search term “(“solar lentigines” OR “solar lentigo” OR “lentigines” OR “lentigo”) AND (“laser”) AND (“cryotherapy”)”.

**Results:**

total of 5 studies (n=13–120, mean age 39–60) comparing lasers (Q-switched Nd:YAG, PDL, argon, CO2, etc.) vs cryotherapy for solar lentigines, mostly using intrapatient randomization, were included. Meta-analysis revealed no significant difference in marked (OR=1.15, 95% CI [-0.42, 2.73], P=0.151, I²=0.6%) or moderate improvement (OR=0.63, 95% CI [-0.01, 1.26], P=0.055, I²=0%), but lasers yielded significantly higher ≥50% improvement in patients (OR=1.36, 95% CI [0.5, 2.21], P=0.002, I²=0.3%), lower mild (OR=-0.96, 95% CI [-1.65, -0.26], P=0.007, I²=0%) and poor response in lesions (OR=-1.72, 95% CI [-3.2, -0.24], P=0.023, I²=0%). Side effects were comparable; heterogeneity remained very low (I²≤0.9%). Four studies showed high risk of bias. One trial demonstrated fractional CO2 or cryotherapy plus 4% pidobenzone significantly outperformed monotherapy.

**Conclusion:**

Laser may be superior to cryotherapy in the treatment of lentigines with comparable side effects among the two groups. However, we recommend further RCTs comparing the two modalities to confirm our findings which had many limitations.

## Introduction

Skin disorders are considered a major issue in the recent world settings with the remarkable improvements in the field of skin care and cosmetics. Lentignies is one of the pigmentary skin lesions causing abnormal skin color mainly in sun-exposed areas in particular the hands and face [1]. Of note, lentigines is the most presented pigmentary skin lesion in a population based study of Abdel-Hafez et al, with a prevalence of 7% among the total population and around two fifth regarding the pigmentary skin lesions only [2].

The most accepted theory of lentigines development is the prolonged exposure of the ultraviolet radiation (UVR) from the sun causing abnormal skin production of melanocytes which produce characteristic abnormal skin color [3]. However, further evidence indicated that environmental factors other than UVR may play an important role in lentigines development [4]. Furthermore, recent evidence suggests that diabetes mellitus is association with lentigines development rather than controls [5].

Treatment of lentigines composed of several strategies including laser, cryotherapy, local ointments and peels. Some clinicians prefers single treatment strategy and others may prescribe combined treatment aiming for more efficacy [6]. Various clinical studies have demonstrated the safety and efficacy of laser therapy compared to cryotherapy; indicating that laser may be effective than cryotherapy in lentigines treatment [7, 8, 9, 10, 11]. However, there is no meta-analysis comparing the two techniques together aiming for choosing the efficient one with the least reported side effects. So in this systematic review and meta-analysis we aimed to study the safety and efficacy of cryotherapy versus laser in lentigines treatment.

## Materials and Methods

This systematic review adhered strictly to the PRISMA guidelines [12]. From database inception through 19 May 2023, investigators conducted a literature search across five electronic databases (Web of Science=33 records, Scopus=79 records, PubMed=28 records, Virtual Health Library=30 records, and Google Scholar=622 records). The search strategy employed the following Boolean string: (“solar lentigines” OR “solar lentigo” OR “lentigines” OR “lentigo”) AND (“laser”) AND (“cryotherapy”). All retrieved records underwent duplicate removal and were subsequently exported to a Microsoft Excel spreadsheet for systematic screening. Two independent authors performed each screening phase (title/abstract screening followed by full-text evaluation) and classified studies as included, excluded, or uncertain. In instances of disagreement, a third author arbitrated to reach consensus. Inclusion criteria: Randomized controlled trials (RCTs) that evaluated the safety and efficacy of laser therapy compared with cryotherapy for the treatment of solar lentigines were eligible for inclusion. Exclusion criteria: Non-RCT designs (including cohort studies, cross-sectional studies, case series, case reports, and review articles), conference abstracts, and narrative literature reviews were excluded.

### Data Extraction

Data extraction was performed independently by two authors using a standardized form. Variables extracted from each eligible study encompassed study identifier, total sample size, percentage of male participants, duration of follow-up, and mean age of participants. Efficacy outcomes were uniformly categorized according to the degree of lesion lightening: (1) marked improvement (75–100% lightening), (2) moderate improvement (50–75% lightening), (3) mild improvement (25–50% lightening), and (4) poor improvement (0–25% lightening). Safety outcomes, including adverse events, were also systematically recorded. Given the heterogeneity in reporting (i.e., outcomes presented per patient or per lesion), data were pooled within each category to avoid biased under- or overestimation of treatment effects.

### Risk of Bias

The Cochrane Risk of Bias Tool for Randomized Trials (RoB 2) was applied to assess methodological quality, as all included studies were RCTs. Each domain was rated as low risk, some concerns, or high risk, with an overall risk-of-bias judgment assigned to every study.

### Statistical Analysis

r language packages of meta was utilized for statistical computations. Effect sizes were calculated as odds ratios (ORs) with corresponding 95% confidence intervals (95% CIs) by pooling event counts and totals from each study. A random-effects model was employed owing to the presence of substantial statistical heterogeneity across included studies [13]. Meta-regression and publication bias assessments (funnel plot asymmetry or Egger’s test) were not performed because fewer than 10 studies contributed to each outcome.

## Results

**Table:** Table1. Characteristics of Included Studies

Study	Year	n	Treatment Site	Interventions Compared	Sessions	Follow-up Period	Outcome Assessment	Efficacy Ranking
Seirafi et al.	2011	22	Face or dorsum of hands	595-nm long-pulse PDL (10 J/cm², 7 mm spot, 1.5 ms, compression handpiece, no cooling) vs Cryotherapy (single freeze-thaw)	1	4 weeks	Physician-rated degree of lesion lightening	PDL superior overall; marked advantage in types III–IV
Dawood et al.	2015	120 (60 per arm)	Not reported	Q-switched Nd:YAG laser vs Cryotherapy (liquid nitrogen, cotton-swab, –20 °C)	8 (every 15 days)	End of treatment	5-point clinical improvement scale	Q-switched Nd:YAG significantly better than cryotherapy
Stern et al.	1994	13 (99 lesions)	Multiple sites	Argon laser (shuttered) vs Low-fluence CO2 laser vs Cryotherapy (liquid nitrogen)	1	Not specified	Rate of substantial lightening and excellent results	Cryotherapy > argon laser > CO2 laser
Todd et al.	2000	27	Dorsal hands	Frequency-doubled Q-switched Nd:YAG vs Krypton laser vs 532-nm diode-pumped laser vs Cryotherapy	1	6 weeks & 12 weeks	Blinded photographic evaluation + patient preference	Q-switched Nd:YAG > krypton > diode-pumped > cryotherapy
Campanati et al.	2016	72	Not reported (solar lentigines)	Pidobenzone 4% topical (adjuvant) + fractional CO2 laser vs Pidobenzone 4% topical (adjuvant) + cryotherapy vs Physical therapy alone	Multiple (details not in abstract)	Not reported	Skin Tone Color Scale (STCS) Visual Analog Scale (VAS) patient impression	Combination therapy > physical therapy alone Cryotherapy + pidobenzone 4% = most effective combination

**Table:** Table2. Safety of Laser Vrsus Cryotherapy

Study ID	Compared groups	Erythema	Scar	Pupura	Pigmentation	Hypopigmentation	Hyperpigmentation
Seirafi-2020-Iran	Laser Cryotherapy	6/22 8/22	0/22 0/22	0/22 0/22	0/22 0/22	-	-
Todd-2000-USA	Laser Cryotherapy	25/75 15/75	1/75 0/75	-	-	2/75 1/75	1/75 0/75

**Table:** Table3. Risk of Bias Among the Included Studies

Study ID	Risk of bias arising from the randomization process	Risk of bias due to deviations from the intended interventions	Risk of bias due to missing outcome data	Risk of bias in measurement of the outcome	Risk of bias in selection of the reported result	Overall risk of bias
Seirafi-2020-Iran	High risk	High risk	Low risk	High risk	Low risk	High risk
Dawood-2020-Pakistan	High risk	High risk	Low risk	High risk	Low risk	High risk
Stern-1989-Israel	Low risk	Some concerns	Low risk	Low risk	Low risk	Some concerns
Todd-2000-USA	High risk	High risk	Low risk	High risk	Low risk	High risk
Campanati-2016-Italy	High risk	High risk	Low risk	High risk	Low risk	High risk

### Study Process

**Figure-1:**
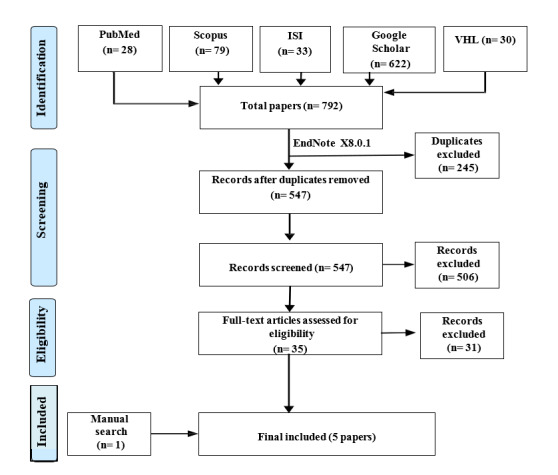
Figure 1. PRISMA flow diagram

We found 792 records from the systematic search in the five databases. Of those, 245 records were considered duplicates. Title and abstract screening of 547 records resulted in 35 eligible full texts for further screening. Finally, we included 4 papers and another paper from manual search methods (Figure-1) [7, 8, 9, 10, 11].

**Figure-2:**
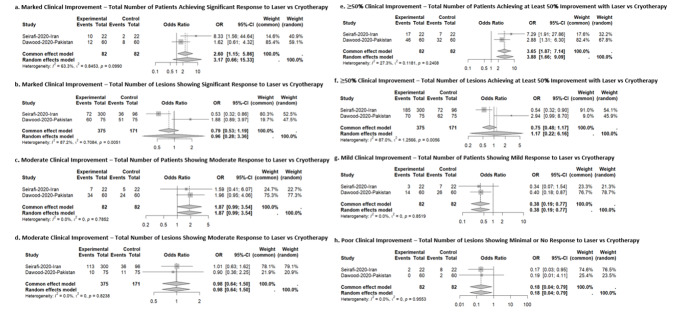
Figure 2. Forest plots of meta-analysis of different study outcomes

### Study Characteristics

The reviewed studies on the treatment of solar lentigines encompassed a geographically diverse patient population from Iran, Pakistan, the USA, Israel, and Italy, as detailed in Table1. Sample sizes ranged from 13 to 120 participants, with a mean or median age typically in the fifth to sixth decade (39–60 years). Only one study (Seirafi et al., 2011, Iran) randomized patients into separate treatment groups comparing 595-nm long-pulse pulsed dye laser (PDL) versus cryotherapy, whereas the remaining four investigations (Dawood et al., 2015, Pakistan; Stern et al., 1994, Israel; Todd et al., 2000, USA; Campanati et al., 2016, Italy) employed intrapatient randomization by allocating different lesions within the same individual to distinct interventions, thereby minimizing inter-subject variability. Interventions predominantly pitted laser modalities—including Q-switched Nd:YAG, argon, low-fluence CO2, frequency-doubled Q-switched Nd:YAG, krypton, 532-nm diode-pumped, and fractional CO2 (the latter combined with 4% pidobenzone topical adjuvant)—against cryotherapy (liquid nitrogen, single or double freeze-thaw cycles), with session numbers varying from a single treatment to eight biweekly sessions. Follow-up durations were heterogeneous: 4 weeks (Seirafi et al.), 8 weeks (Stern et al.), 12 weeks (Todd et al. and Campanati et al.), and 16 weeks (Dawood et al.), while one study did not specify follow-up length. Outcome measures included physician-rated lesion lightening, 5-point clinical improvement scales, blinded photographic evaluations, patient preference, and validated instruments such as the Skin Tone Color Scale (STCS) and Visual Analog Scale (VAS). Efficacy rankings consistently favored laser-based approaches over cryotherapy in most trials, with notable superiority of Q-switched Nd:YAG (Dawood et al., Todd et al.), PDL in types III–IV lentigines (Seirafi et al.), and fractional CO2 plus pidobenzone as the most effective combination (Campanati et al.), although cryotherapy occasionally outperformed certain lasers (Stern et al.). Efficacy In the meta-analysis, there was no significant difference between laser and cryotherapy regarding marked improvement in the total number of patients (random-effects OR = 1.15, 95% CI [-0.42, 2.73], [P=0.151, I²=0.6%; Figure-2a) or in the total number of lesions (random-effects OR=-0.04, 95% CI [-1.28, 1.21], P=0.954, I²=0.9%; Figure-2b). Similarly, moderate improvement did not differ significantly between the two interventions in either patients (random-effects OR=0.63, 95% CI [-0.01, 1.26], P=0.055, I²=0%; Figure-2c) or lesions (random-effects OR=-0.02, 95% CI [-0.44, 0.4], P=0.935, I²=0%; Figure-2d). For ≥50% improvement, laser was significantly more effective than cryotherapy in patients (random-effects OR=1.36, 95% CI [0.5, 2.21], P=0.002, I²=0.3%; Figure-2e), but not in lesions (random-effects OR=0.16, 95% CI [-1.5, 1.82], P=0.853, I²=0.9%; Figure-2f). Regarding mild improvement, laser showed a significant reduction compared to cryotherapy in patients (random-effects OR=-0.96, 95% CI [-1.65, -0.26], P=0.007, I²=0%; Figure-2g). Finally, poor improvement was significantly lower in lesions treated with laser than with cryotherapy (random-effects OR=-1.72, 95% CI [-3.2, -0.24], P=0.023, I²=0%; Figure-2h). Across outcomes, heterogeneity was low (I²≤0.9% for all comparisons), indicating consistency among studies.

### Side Effects

The prevalence of side effects was comparable between the two groups (Table2). We did not conduct meta-analysis as the prevalence was reported in one study in each side effect, or reported in one study with number of patients and the other in number of lesions which cannot be pooled together.

### Combined Treatment Versus Individual Treatment

One study reported that the combined treatment of pidobenzone 4% + cryotherapy and pidobenzone 4% + laser were associated with a significant improvement in the Skin Tone Color Scale and the Visual Analog Scale rather than cryotherapy or laser alone [11].

### Risk of Bias

Risk-of-bias assessment revealed four studies at high risk and only one (not specified) raising some concerns, primarily due to limitations in randomization, blinding, and reporting consistency (Table3).

## Discussion

In our study, we found that laser therapy was better than cryotherapy in achieving at least 50% improvement in lentigines patients. However, the comparison did not reveal statistical significance when comparing the two therapies regarding marked (75%-100%) or moderate (50%-75%) improvement alone. Furthermore, laser was significantly associated with decreased rates of mild and poor improvement.

Our study is similar to the results of the RCT of Seirafi et al., where 45.5% and 31.8% of patients who received laser had marked and moderate improvement compared to 9% and 22.7% of patients who received cryotherapy. It is interesting that laser therapy resulted in ≥ 51% improvement in three quadrants of patients compared to only one third of patients who received cryotherapy [7]. The same results were obtained from the RCT of Dawood et al, which indicated that three quadrants of patients allocated to laser achieved ≥51% improvement rather than half of patients allocated to cryotherapy group [8]. Such results explain why laser therapy is inferior to cryotherapy in inducing poor and mild improvement in our study.

In the other two RCTs who investigated the effect of laser and cryotherapy regarding the total number of lesions, the improvement rates were nearly similar between the two treatment groups [9, 10]. We did not combine the two RCTs [9, 10], with the other RCTs because the reported results differs in the reported estimates which will progress to results bias as the number, location and the severity of lesions may have a significant impact on the treatment response [14].

It is worth noting that combined treatment of lentigines is better than single treatment [14]. We found that only one study that combined pidobenzone 4% to laser or cryotherapy. The results of this study demonstrated the superiority of combined treatment rather than single treatment option; however it did not demonstrate which treatment modality (laser or cryotherapy) is better when combined with another treatment agent [11].

The data regarding safety in our included papers are scarce and reported in only two RCTs; however there were no serious adverse events in the treatment groups, laser or cryotherapy. However, Todd and colleagues reported that erythema is more common in laser group in particular krypton laser than cryotherapy which may be attributed to the nature of laser in inducing such side effect [10]. Moreover, the rates of erythema were comparable between the two groups revealed by the trial of Seirafi et al. [7].

Our findings align closely with subsequent evidence from broader systematic reviews and targeted clinical studies. Mardani et al. (2025) [15], in a review of 41 trials encompassing 3234 patients, reported cryotherapy success rates of only 37%–71.4%, markedly lower than those of most laser modalities (pulsed dye laser 27%–57%, intense pulsed light 74.6%–90%, Q-switched lasers 36.36%–76.6%, picosecond lasers 67.9%–93.02%), while explicitly noting that cryotherapy was associated with more severe adverse events, particularly post-inflammatory hyperpigmentation (PIH). This safety disadvantage was corroborated by Seirafi et al. (2011) [7], whose split-face/hand RCT in skin types II–IV showed that long-pulse 595-nm PDL with compression produced superior lightening (statistically significant in types III–IV) and elicited only minimal erythema, whereas cryotherapy exclusively caused PIH. Furthermore, the exploratory work by Szymańczyk et al. (2021) [16] on a novel 450-nm blue laser achieved complete clearance in facial and dorsal hand lentigines, locations where cryotherapy historically performs relatively better, yet resulted in unsatisfactory scarring on the trunk and forearms, showing the site-specific limitations of non-selective thermal damage inherent to cryotherapy.

Despite that we included all the available evidence regarding the safety and efficacy of laser versus cryotherapy in lentigines treatment. Our results must be confirmed with new published papers in this topic as we faced many limitations. Firstly, we experienced significant heterogeneity among the included studies. Secondly, the follow up was short in most of the included studies. Thirdly, few studies reported treatment side effects. Fourthly, only one study reported the effect of combined therapy to laser or cryotherapy without indicating the superiority of this combination regarding the two modalities. Fifthly, most of our studies were at high risk of bias.

## Conclusion

Laser may be superior to cryotherapy in the treatment of lentigines with comparable side effects among the two groups. However, we recommend further RCTs comparing the two modalities to confirm our findings which had many limitations.

## Conflict of Interest

The authors declare no conflict of interest.

## AI Disclosure Statement

During the preparation of this manuscript, the authors used ChatGPT, OpenAI company for language editing, grammar improvement, and liboberry.com for reference management. After its use, the authors thoroughly reviewed, verified, and revised all AI-assisted content to ensure accuracy and originality. The authors take full responsibility for the integrity and final content of the published article.
